# Global Gene Expression Analysis of Canine Cutaneous Mast Cell Tumor: Could Molecular Profiling Be Useful for Subtype Classification and Prognostication?

**DOI:** 10.1371/journal.pone.0095481

**Published:** 2014-04-18

**Authors:** Mery Giantin, Anna Granato, Chiara Baratto, Laura Marconato, Marta Vascellari, Emanuela M. Morello, Antonella Vercelli, Franco Mutinelli, Mauro Dacasto

**Affiliations:** 1 Dipartimento di Biomedicina Comparata e Alimentazione, Università di Padova, Legnaro (Padova), Italy; 2 Istituto Zooprofilattico Sperimentale delle Venezie, Legnaro (Padova), Italy; 3 Centro Oncologico Veterinario, Sasso Marconi, Bologna, Italy; 4 Dipartimento di Scienze Veterinarie, Università di Torino, Grugliasco (Torino), Italy; 5 Ambulatorio Veterinario Associato, Torino, Italy; The Ohio State University/OARDC, United States of America

## Abstract

Prognosis and therapeutic management of dogs with cutaneous mast cell tumors (MCTs) depend on clinical stage and histological grade. However, the prognostic value of this latter is still questionable. In the present study, MCT transcriptome was analyzed to identify a set of candidate genes potentially useful for predicting the biological behavior of MCTs. Fifty-one canine MCT biopsies were analyzed. Isolated and purified total RNAs were individually hybridized to the Agilent Canine V2 4x44k DNA microarray. The comparison of reference differentiated and undifferentiated MCT transcriptome revealed a total of 597 differentially expressed genes (147 down-regulated and 450 up-regulated). The functional analysis of this set of genes provided evidence that they were mainly involved in cell cycle, DNA replication, p53 signaling pathway, nucleotide excision repair and pyrimidine metabolism. Class prediction analysis identified 13 transcripts providing the greatest accuracy of class prediction and divided samples into two categories (differentiated and undifferentiated), harboring a different prognosis. The Principal Component Analysis of all samples, made by using the selected 13 markers, confirmed MCT classification. The first three components accounted for 99.924% of the total variance. This molecular classification significantly correlated with survival time (*p* = 0.0026). Furthermore, among all marker genes, a significant association was found between mRNA expression and MCT-related mortality for FOXM1, GSN, FEN1 and KPNA2 (*p*<0.05). Finally, marker genes mRNA expression was evaluated in a cohort of 22 independent samples. Data obtained enabled to identify MCT cases with different prognosis. Overall, the molecular characterization of canine MCT transcriptome allowed the identification of a set of 13 transcripts that clearly separated differentiated from undifferentiated MCTs, thus predicting outcome regardless of the histological grade. These results may have clinical relevance and warrant future validation in a prospective study.

## Introduction

Nowadays, molecular profiling technologies provide the potential to comprehend relevant biological networks underlying the cellular and molecular origin of cancer as well as to tailor medical care, both at tumor and patient levels [1], [2]. Gene expression profiling has shown a great potential in cancer research, providing a detailed view on the molecular changes involved in tumor progression, leading to a better understanding of the pathophysiological process, discovering new prognostic markers and novel therapeutic targets [1].

An increasing number of –omic oncologic studies have been recently published in the dog, focusing on mammary tumor [3], [4], [5], [6], hemangiosarcoma [7], osteosarcoma [8], [9], [10], lymphoma [11], [12], urinary bladder cancer [13], histiocytic sarcoma [14] and melanoma [15]. Conversely, the transcriptome of canine cutaneous mast cell tumor (MCT) has not been characterized so far, although MCTs are frequent tumors in dogs, accounting for approximately 6% of all canine neoplasms and 20% of all skin tumors [16], [17].

Unlike human cutaneous mastocytosis, that is rare and benign [18], the disease course of canine MCT may be aggressive [17]. Currently, therapy is mainly guided by histological grade and clinical stage, which have prognostic relevance [19], [20]. Nevertheless, the prognostic value of the histological grading is still questionable, particularly for Patnaik grade 2 (G2) MCTs. Indeed, while the biological behavior for Patnaik-G1 and Patnaik-G3 MCTs can generally be anticipated, the prognosis for Patnaik-G2 MCTs is variable [17]. In addition to the unpredictable behavior of Patnaik-G2 MCTs, histopathological grading is subjective, resulting in grading variations among pathologists [21], [22]. In 2011, a new grading system was proposed by Kiupel and coauthors, aiming at improving concordance among pathologists [20]. This two-tier histologic grading system was demonstrated to be more accurate at predicting metastasis development and tumor mortality than the Patnaik system [20]. Nevertheless, approximately 15% of dogs with Kiupel low grade MCTs have nodal involvement at presentation (Marconato, personal data), indicating that also this grading system possesses some gaps.

The aim of the present study was to characterize the MCT transcriptome by using DNA microarray technology, in order to define a fingerprint of aggressiveness that could predict the biological behavior, possibly overcoming the pitfalls of histological grading.

## Materials and Methods

### Ethics Statements

Animal care, surgery and post surgery were carried out in accordance with good veterinary practices; dogs were under the care of licensed veterinarians and participation in the study did not influence decisions of care. According to the Italian law (D. Lgs. n. 116/92), an Institutional Animal Care and Use Committee approval number and date of approval for the study are not requested for both academia and private practice. Only a written informed consent is needed to conduct a clinical trial.

### Tumor Samples

In this prospective study, seventy-three histologically confirmed samples of spontaneous canine cutaneous MCTs were collected from veterinary clinics of Northern Italy throughout the years 2007–2013, during routine diagnostic or therapeutic surgical procedure and after written informed consent of the owner. Aliquots from the central part of the tumor mass (up to 100 mg each) were aseptically collected, immediately stored in RNAlater solution (Life Technologies, Foster City, CA) and kept at –20°C until use.

For each included case, the following data were retrieved from medical records: breed, age, sex, grade according to Patnaik’s system and Kiupel’s [19], [20], type of treatment (surgery, radiation therapy, chemotherapy, tyrosine kinase inhibitors or a combination of these), survival time, and cause of death.

MCTs from 51 dogs yielded high quality total RNA and passed the quality control for gene expression profiling on DNA microarrays. The remaining 22 samples were used in quantitative Real Time PCR (qPCR) confirmatory analyses.

For sample class prediction, 18 out of the 51 samples used for gene expression profiling were chosen as “reference samples” on the basis of Patnaik and Kiupel histological classification and mitotic index; these “reference samples” included 13 “differentiated MCTs” (Patnaik-G1 or G2 and Kiupel low grade [L], with a mitotic index ≤5), and 5 “undifferentiated MCTs” (Patnaik-G2 or G3, Kiupel high grade [H], with a mitotic index >5). The choice of MCT reference samples was based on a 100% concordance among all pathologists.

### RNA Isolation and Purification

Total RNA was isolated from tissue specimens as previously described [23] and subsequently purified using the RNeasy Mini kit (Qiagen, Milan, Italy), according to the manufacturer’s instructions. To avoid genomic DNA contaminations, on-column DNase digestion with the RNase-free DNase set (Qiagen, Milan, Italy) was performed. Total RNA concentration was determined using the NanoDrop ND-1000 UV-Vis spectrophotometer (NanoDropTechnologies, Wilmington, DE), and quality was measured by using the 2100 Bioanalyzer and RNA 6000 Nano kit (Agilent Technologies, Santa Clara, CA). All 51 samples included in the gene expression profiling experiment were suitable for microarray analysis based on RNA quality (RIN≥7.0).

### RNA Amplification, Labeling and Array Hybridization

Sample amplification, labeling and hybridization were performed following the Agilent One-Color Microarray-Based Gene Expression Analysis protocol. Briefly, for each individual sample, 100 ng of total RNA were linearly amplified and labeled with Cy3-dCTP using Agilent Low Input Quick Amp Labeling kit (Agilent Technologies, Santa Clara, CA) according to the manufacturer’s instructions. A mixture of 10 different viral poly-adenylated RNAs (Spike-In Mix, Agilent Technologies, Santa Clara, CA) was added to each RNA sample before amplification and labeling, to monitor microarray analysis work-flow. Labeled cRNA was purified with RNeasy Mini Kit (Qiagen, Milan, Italy), and sample concentration and specific activity (pmol Cy3/µg cRNA) were measured with a NanoDrop ND-1000 UV-Vis spectrophotometer. A total of 1.65 micrograms of labeled cRNA was fragmented by using the Gene Expression Hybridization kit (Agilent, Santa Clara, CA) following the manufacturer’s instructions. A volume of 100 µL of hybridization solution was then dispensed in the gasket slide and assembled to the microarray slide, with each slide containing four arrays. Canine-specific oligo-arrays (Canine V2 4x44k G2519F, Design ID 021193, Agilent Technologies, Santa Clara, CA) were used. Slides were incubated for 17 h at 65°C in an Hybridization Oven (Agilent Technologies, Santa Clara, CA), subsequently removed from the hybridization chamber, quickly submerged in GE Wash Buffer 1 to disassembly slides and then washed in GE Wash Buffer 1 for approximately 1 minute, followed by one additional wash in pre-warmed (37°C) GE Wash Buffer 2. Hybridized slides were scanned at 5 µm resolution using a G2565BA DNA microarray scanner (Agilent Technologies, Santa Clara, CA). Default settings were modified in order to scan the same slide twice at two different sensitivity levels (XDR Hi 100% and XDR Lo 10%). Total RNA of 4 samples was labeled twice and hybridized separately in different slides to generate technical replicates.

Microarray data have been deposited in NCBI's Gene Expression Omnibus, and are accessible through GEO Series accession number GSE50433.

### Normalization of Microarray Data

The two linked images generated from the scanned slide were analyzed together, data were extracted and background subtracted by using the standard procedures contained in the Agilent Feature Extraction Software version 9.5.1. All 51 samples were then normalized together in a single run to avoid potential biases. Normalization procedures were performed by means of the R statistical software (http://www.r-project.org), and by using Spike-In control intensities to identify the best normalization procedure. Normalization using limma package always yielded better results than cyclic loess normalization, thus quantile-normalized data were used in all subsequent analysis. After normalization, spike intensities are expected to be uniform across the experiments of a given dataset. All control features and Spike-in were excluded from subsequent analyses. Missing values (probes with Feature Extraction flag equal to 0) were imputed by using the microarray data analysis tool TIGR Multiple Array Viewer (TMEV) [24].

### Statistical Analyses

To identify differentially expressed genes between “reference samples” (differentiated and undifferentiated MCTs), a two-class unpaired test was implemented in the program SAM (Significance Analysis of Microarrays) release 4.0 [25], enforcing a False Discovery Rate (FDR) of 1% and a –fold change (FC) of 2.

Gene expression data from 51 MCTs were analyzed to evaluate the ability to classify MCT samples with a reduced set of informative markers, by using a statistical approach for class prediction implemented in the Prediction Analysis of Microarrays (PAM) software, available online at http://www-stat.stanford.edu/~tibs/PAM. This software uses the method of nearest shrunken centroids to find out the minimal set of genes that provides the greatest accuracy of class prediction. The program first performs a discriminant analysis on “known” samples (Training Sample Set, in this study these known samples were the 18 reference samples as previously described) to choose the smallest panel of genes providing the greatest accuracy of class prediction with the smallest misclassification error. Subsequently, the program uses the same panel of genes to predict a class for a set of “unknown” samples (in this study represented by 33 of 51 MCTs, for whose grading the pathologists did not reach a consensus).

To confirm the correct choice of reference samples and PAM class prediction results, hierarchical clustering (HCL) and principal component analysis (PCA) on gene expression data were carried out by using the TMEV suite.

All other statistical tests (linear regression, non-parametric Spearman correlation analysis and Mann-Whitney test) were carried out with the GraphPad Prism 5 software (San Diego, CA, USA). Statistical significance was set at *p*<0.05.

### Functional Annotation

The functional annotation analysis of differentially transcribed genes was performed using the Database for Annotation, Visualization and Integrated Discovery (DAVID) web-server v.6.7 (http://david.abcc.ncifcrf.gov) [26]. Gene ontology (GO) terms and Kyoto Encyclopedia of Genes and Genomes (KEGG) pathways included in the DAVID knowledgebase were considered. The following parameters were respectively used for GO Biological Process and KEGG pathways: gene count 3, ease 0.05; gene count 4, ease 0.05.

### Quantitative Real Time PCR (qPCR)

Thirteen target genes selected through class prediction analysis and three internal control genes (ICGs) were chosen for qPCR amplification. For each transcript, gene-specific primers that encompassed one intron and the most appropriate UPL probe were designed by using the Universal Probe Library (UPL) Assay Design Centre web service (Roche Diagnostics, Mannheim, Germany). Putative intron-exon boundaries were inferred from the UCSC Genome Browser and Ensembl Genome Browser Databases (http://genome.ucsc.edu and www.ensembl.org).

First-strand cDNA was synthesized from 1 µg of total RNA using the High Capacity cDNA Reverse Transcription Kit (Life Technologies, Carlsbad, CA) according to the manufacturer’s protocol and stored at −20°C until use. Primers specificity was evaluated either in silico by means of the BLAST tool than experimentally by Power SYBR Green I (Life Technologies, Carlsbad, CA) amplification and melting curve analysis. qPCR reactions (10 µL final volume) consisted of 1X LightCycler 480 Probe Master (Roche Applied Science, Indianapolis, IN), 300 or 600 nM forward and reverse primers (Eurofins MWG Operon, Ebersberg, Germany) according to the assay set-up, 200 nM human UPL probe and 2.5 µL of 1∶100 diluted cDNA. qPCR analysis was performed in duplicate in a LightCycler 480 Instrument (Roche Applied Science, Indianapolis, IN) using standard PCR conditions (an activation step at 95°C for 10 minutes; 45 cycles at 95°C for 10 seconds and at 60°C for 30 seconds; a cooling step at 40°C for 30 seconds) and LightCycler 480 clear plates (Roche Applied Science, Indianapolis, IN). No-template controls and no-reverse transcription controls were included on each plate. Calibration curves, using a 3-fold serial dilution of a cDNA pool, were performed. ICGs assay parameters were comparable to that of the target genes; moreover, no statistically significant differences were observed in their expression profile between differentiated and undifferentiated MCT samples. Data were analyzed with the LightCycler480 software release 1.5.0 (Roche Applied Science, Indianapolis, IN) by using the second derivative method. Messenger RNA relative quantification was performed by using the ΔΔCt method [27], the arithmetic mean of the three selected ICGs and a cDNA pool comprehending two external MCT samples as calibrator.

Twenty-two MCT samples not processed on the array were chosen for qPCR confirmatory analysis. All thirteen transcripts selected with class prediction analysis (PAM analysis) were amplified in duplicate as reported above. RQ values were finally analyzed by using Multid-Genex software [28]. Clustering and PCA were performed, adopting the following setting: mean center scaling, Ward’s algorithm and Manhattan distance.

### Survival Analysis

Survival time was defined as the interval from date of surgery to death and was investigated by means of Kaplan–Meier survival analysis, stratified by MCT molecular classification (differentiated and undifferentiated MCTs). The log rank test was used to compare survival time between groups.

For each selected target gene, a receiver-operator characteristic (ROC) curve was created. For areas under curve >0.5, the cut-off value (in terms of relative quantification value, RQ) that better discriminated MCT associated with patient mortality was determined. On the basis of the chosen cut-off value, sensitivity and specificity parameters, with 95% confidence intervals, were calculated. All statistical tests were carried out in GraphPad Prism 5 software (San Diego, CA, USA). Statistical significance was set at *p*<0.05.

## Results

### Animals

Dogs characteristics are shown in Table S1, including the 2 population subsets (GEP cohort, n = 51, and dogs not on array, n = 22). Histological grading and mitotic index of reference samples used for class prediction analysis are shown in Table S2. All included dogs received some form of treatment, consisting of surgery (n = 54), surgery and systemic treatment (n = 12), radiation therapy and systemic treatment (n = 3), systemic treatment (n = 3), and a combination of surgery, radiation therapy and systemic treatment (n = 1).

### DNA Microarray Validation

DNA microarray experiments were individually performed in 51 MCT samples, characterized by a RIN≥7. Technical replicates (labeling and hybridization) were conducted for 4 samples arbitrarily chosen. Linear regression analysis of the entire dataset for each technical replicate revealed a r^2^ ranging between 0.973 and 0.993 with a *p*<0.001. Non parametric Spearman correlation analysis of all samples considering RQ values obtained by qPCR target gene amplification and corresponding normalized DNA microarray data showed positive and significant results for all validated genes (Table 1).

**Table 1:** DNA microarray validation: Spearman correlation analysis of normalized DNA microarray data and corresponding qPCR results for the whole set of samples.

Target gene	Spearman R
CCNB2 (probe 1)	0.9306***
CCNB2 (probe 2)	0.9281***
CCNB2 (probe 3)	0.9103***
CDC20	0.9158***
CDCA8	0.9241***
CENPP	0.7348***
FEN1	0.7797***
FOXM1 (probe 1)	0.8014***
FOXM1 (probe 2)	0.8571***
GSN (probe 1)	0.8228***
GSN (probe 2)	0.9256***
GSN (probe 3)	0.9059***
KPNA2 (probe 1)	0.5392***
KPNA2 (probe 2)	0.6434***
NUF2 (probe 1)	0.8869***
NUF2 (probe 2)	0.9538***
NUF2 (probe 3)	0.9453***
NUSAP1	0.8851***
PRC1 (probe 1)	0.9650***
PRC1 (probe 2)	0.9439***
RAD51	0.9075***
UBE2S (probe 1)	0.5951***
UBE2S (probe 2)	0.7484***
UBE2S (probe 3)	0.7752***

***p<0.001; statistical analysis was performed for each target gene-specific probe available on the array.

### Gene Expression Profiling of Reference Samples

The transcriptome of reference samples, e.g., differentiated (n = 13) and undifferentiated (n = 5) MCT samples was compared by using the SAM program. The unpaired t-test with FDR of 1% and FC of 2 revealed a total of 597 differentially expressed genes (DEGs, 147 down-regulated and 450 up-regulated). The complete list of DEGs and corresponding FC is reported in Tables S3–S4. To explore the functional significance of the observed differences, enrichment analysis of up- and down-regulated genes was carried out by using GO Biological Processes and KEGG Pathways analysis through DAVID web-server. Among the 597 DEGs, only 352 had a unique gene identity that could be assigned to GO Biological Process and KEGG Pathway. The following GO Biological Processes were found to be differentially regulated: M-phase, cell cycle phase, cell cycle process, cell division and cell cycle (Table 2). The GO Biological Process definition for DEGs partially reflected what was described for KEGG pathways: cell cycle, DNA replication, oocyte meiosis, progesterone-mediated oocyte maturation, p53 signaling pathway, nucleotide excision repair, pyrimidine metabolism and steroid biosynthesis.

**Table 2:** DAVID functional annotation of the complete list of differentially regulated genes between differentiated and undifferentiated MCTs.

Category	Term	Count	*p* value	Fold Enrichment
GO_BP_FAT	GO:0000279∼M phase	3	5.00E-03	2.28E+01
GO_BP_FAT	GO:0022403∼cell cycle phase	3	7.93E-03	1.83E+01
GO_BP_FAT	GO:0022402∼cell cycle process	3	9.62E-03	1.66E+01
GO_BP_FAT	GO:0051301∼cell division	3	1.15E-02	1.52E+01
GO_BP_FAT	GO:0007049∼cell cycle	3	2.02E-02	1.14E+01
KEGG_PATHWAY	cfa04110:Cell cycle	27	1.14E-20	1.06E+01
KEGG_PATHWAY	cfa03030:DNA replication	11	9.04E-10	1.50E+01
KEGG_PATHWAY	cfa04114:Oocyte meiosis	12	3.70E-06	5.88E+00
KEGG_PATHWAY	cfa04914:Progesterone-mediated oocyte maturation	9	1.41E-04	5.67E+00
KEGG_PATHWAY	cfa04115:p53 signaling pathway	8	1.75E-04	6.49E+00
KEGG_PATHWAY	cfa03420:Nucleotide excision repair	6	2.19E-03	6.33E+00
KEGG_PATHWAY	cfa00240:Pyrimidine metabolism	7	6.36E-03	4.10E+00
KEGG_PATHWAY	cfa00100:Steroid biosynthesis	4	6.69E-03	9.92E+00

GO, Gene Ontology; BP: Biological Process; *p* value: modified Fisher exact P value calculated by DAVID software;

Fold Enrichment defined as the ratio of the two proportions: input genes involved in a biological process and the background information.

**Figure 1:**
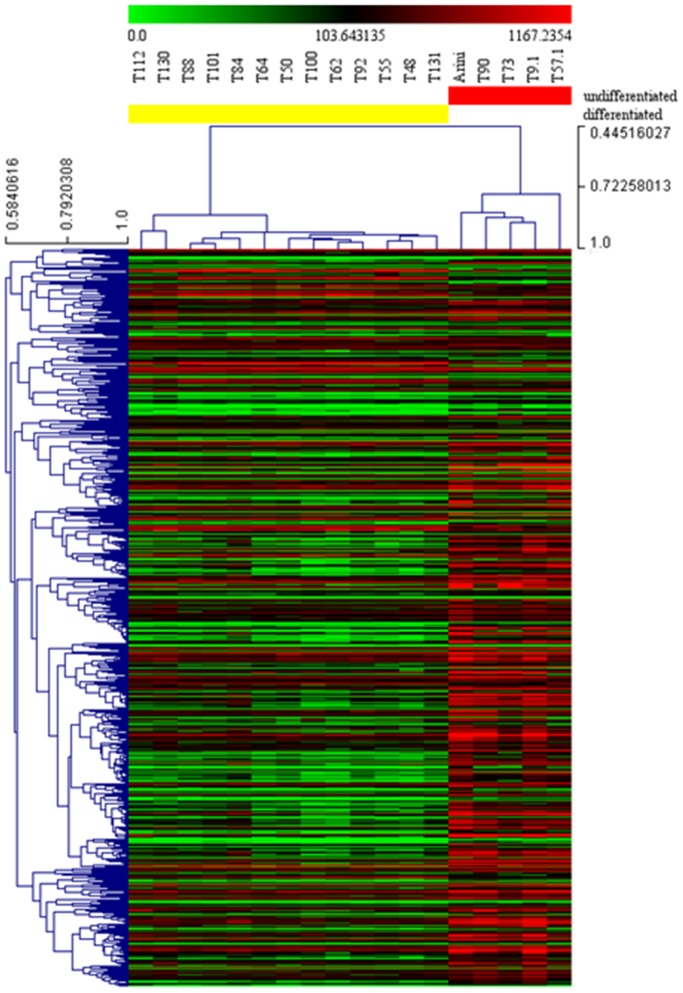
Hierarchical clustering and heat map of differentiated and undifferentiated reference samples. Hierarchical clustering was performed using gene expression data of 597 differentially expressed genes obtained through the comparison of reference samples (13 differentiated and 5 undifferentiated mast cell tumors) with SAM, fixing a fold change of 2 as well as a False Discovery Rate of 0.01 as parameters. Red and green indicates up- and down-regulated genes relative to the mean expression in all samples, respectively. Samples were hierarchically clustered into differentiated (left, yellow) and undifferentiated (right, red) and based on the Pearson correlation coefficients and average linkage clustering. Genes were hierarchically clustered based on Pearson correlation coefficients and average linkage clustering. Units of the bar legend: absolute values.

**Figure 2:**
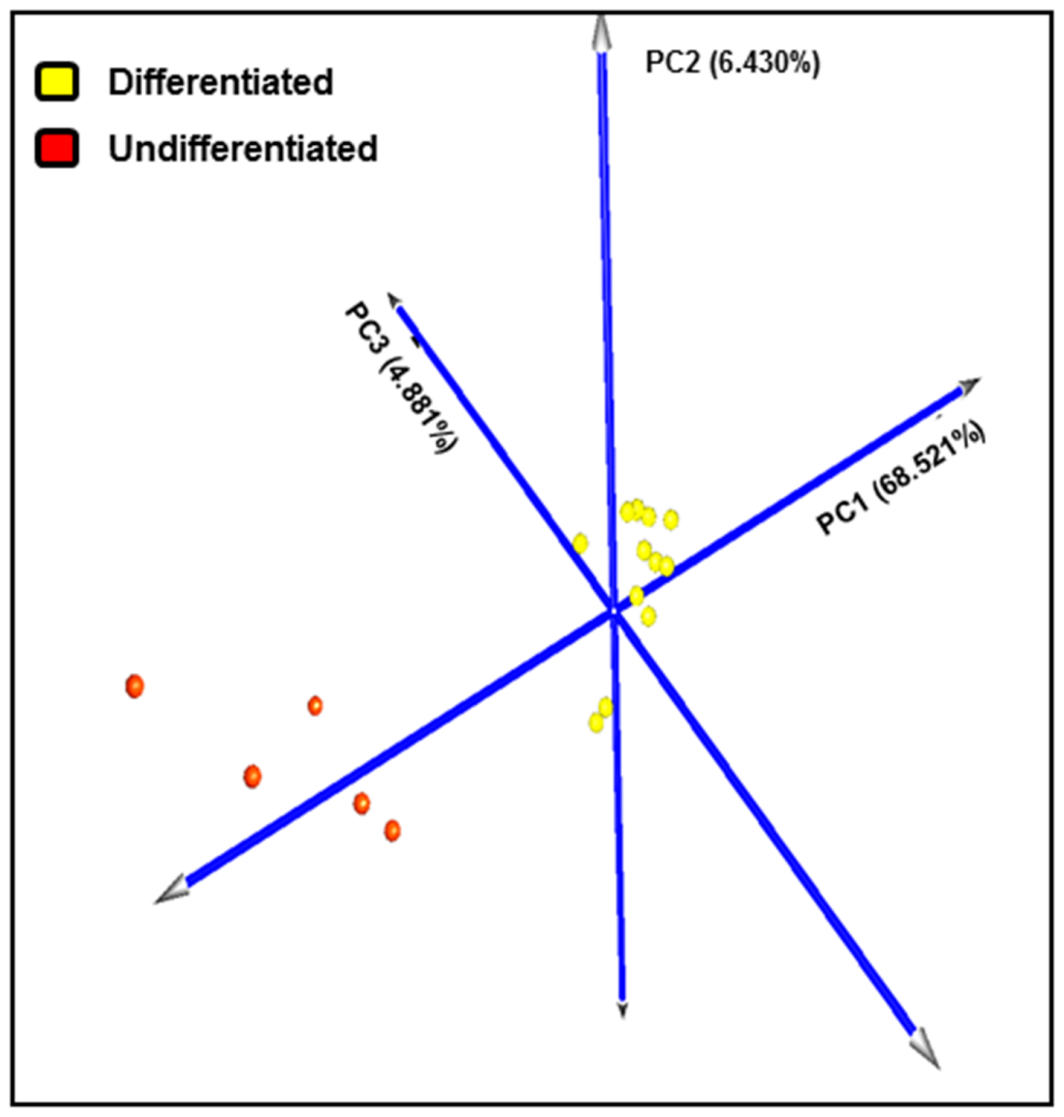
Principal component analysis of differentiated and undifferentiated reference samples. Analysis was performed using gene expression data of 597 differentially expressed genes obtained through the comparison of reference samples (13 differentiated and 5 undifferentiated mast cell tumors) with SAM, fixing a fold change of 2 as well as a False Discovery Rate of 0.01 as parameters. Each colored sphere corresponds to a reference sample (differentiated mast cell tumors are indicated in yellow, while undifferentiated ones in red). The value of each principal component is reported on the graph. The sum of the three principal components accounted to the 79.883% of the total variance.

DEGs dataset obtained through the transcriptome comparison of differentiated and undifferentiated reference samples was analyzed by HCL and PCA. Both HCL tree and PCA clearly identified, without overlapping, two main groups, being attributable to differentiated and undifferentiated reference samples (Figures 1–2). The first three components accounted for a substantial fraction (79.883%) of the total variance. This result confirmed that the samples chosen as reference samples were good as Training Sample Test for class prediction analysis.

### Class Prediction Analysis

**Figure 3:**
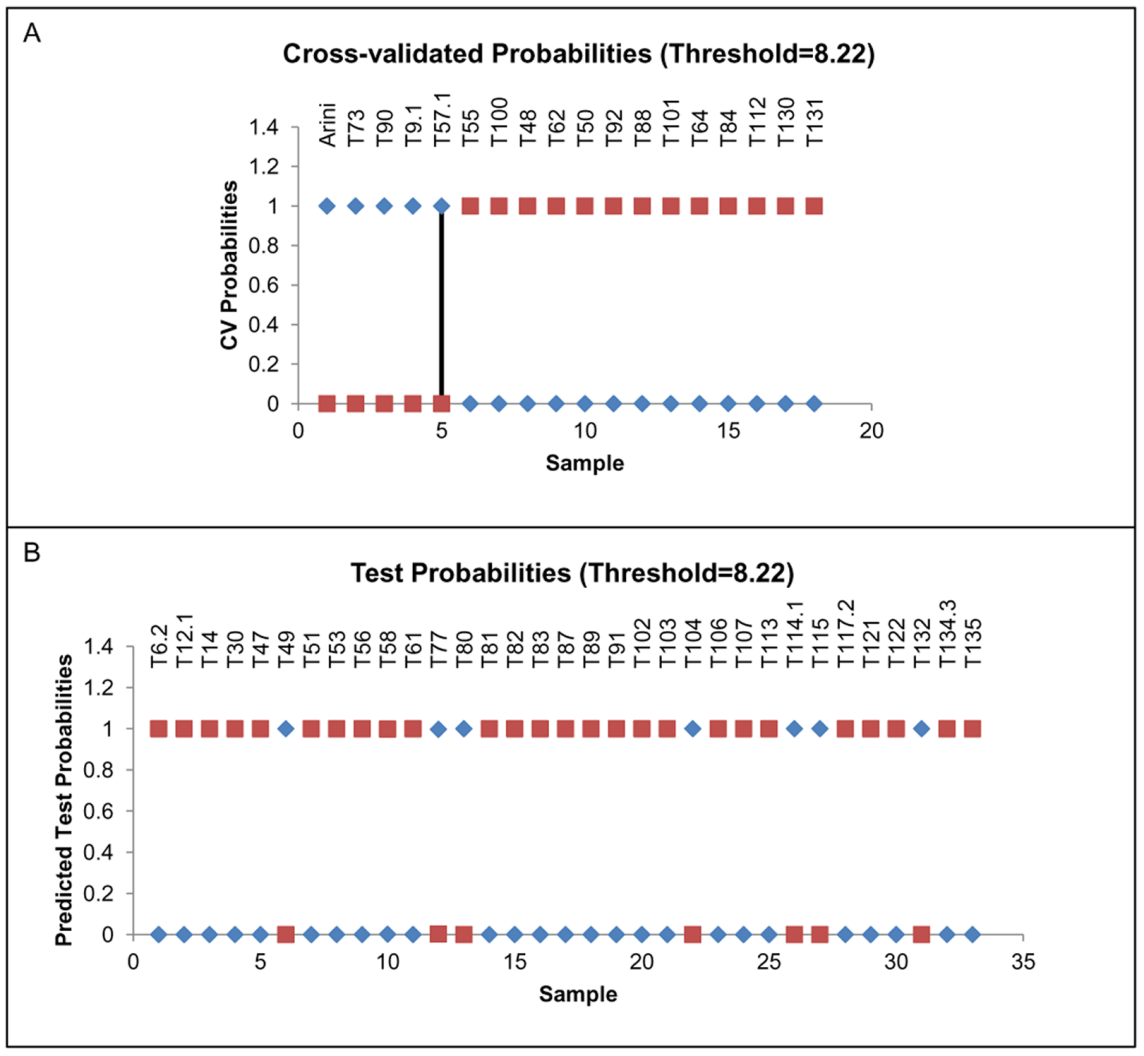
Class prediction analysis. Plot of cross-validated probabilities and test probabilities for sample classification. (A) On x-axis individual reference samples: 1–5 undifferentiated mast cell tumor samples, 6–18 differentiated mast cell tumor samples; on y-axis the probability of being classified as undifferentiated (blue rhombus) or differentiated (red squares). (B) On x-axis individual unknown samples; the probability of being classified as undifferentiated (blue rhombus) or differentiated (red squares).

Gene expression data from all 51 MCT samples were analyzed to evaluate the ability to classify “unknown” samples with a reduced set of informative markers. Differentiated and undifferentiated reference MCTs were used as “known” samples. The nearest shrunken centroid analysis implemented in the PAM program was effective in discriminating between differentiated and undifferentiated reference samples with a 100% accuracy (misclassification error of 0), thereby selecting 13 transcripts. This minimal gene set was then used in the cross-validation procedure yielding a probability of correct identification (about 100%) for all 33 remaining samples (Figure 3). The class prediction analysis allowed the identification, among the unknown samples, of a total of 7 undifferentiated and 26 differentiated MCTs.

**Figure 4:**
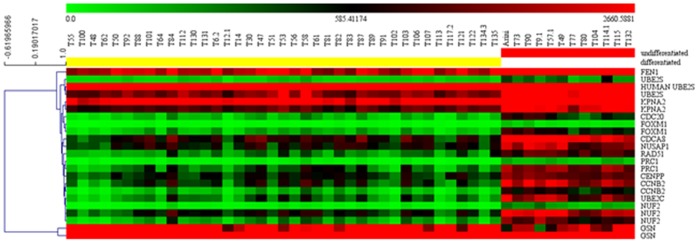
Gene expression profiling of marker genes in differentiated and undifferentiated MCT samples. Heat map and hierarchical clustering of all samples analyzed by DNA microarray using probes referable to the transcripts identified by class prediction analysis. Red indicates up-regulated and green down-regulated genes relative to the mean expression in all samples. For display purposes, samples in each class (differentiated and undifferentiated) were clustered separately and arranged from differentiated (left, yellow) to undifferentiated (right, red). Genes were hierarchically clustered separately based on the Pearson correlation coefficients and average linkage clustering. Units of the bar legend: absolute values.

**Figure 5:**
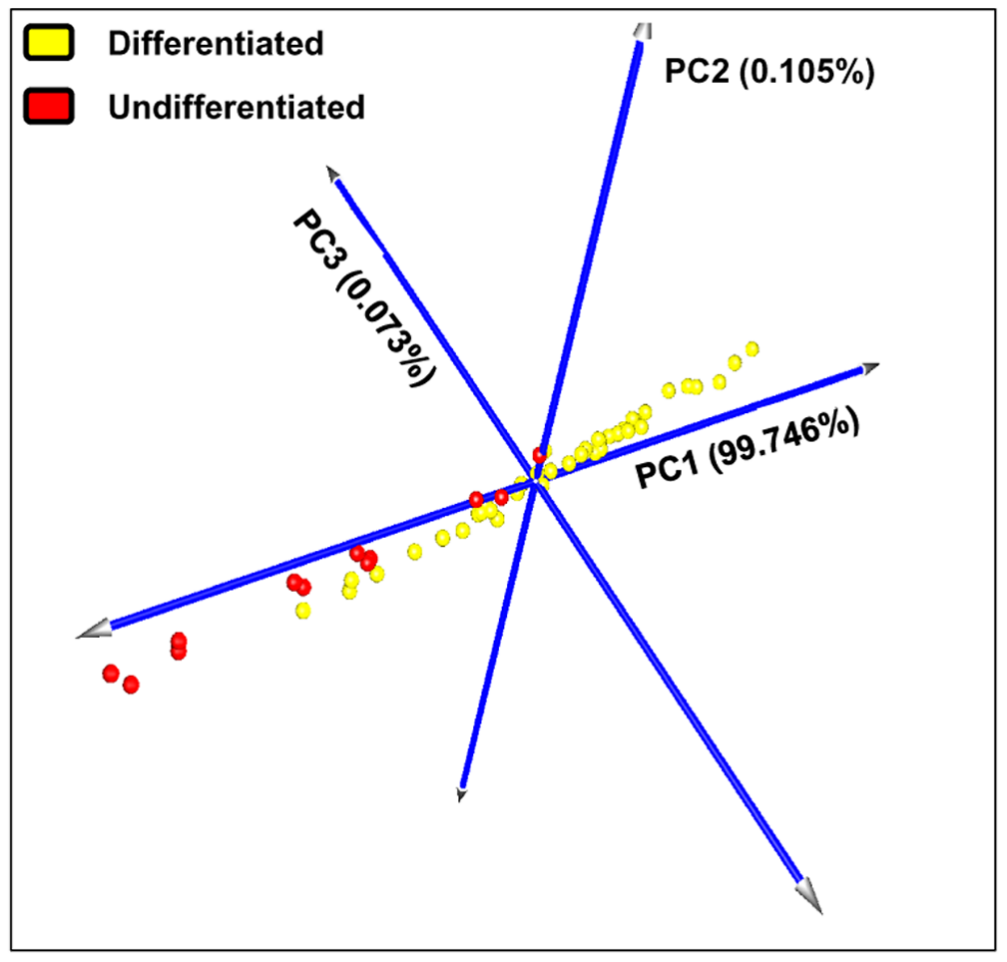
Principal component analysis of array-processed samples using marker gene data. Each colored sphere corresponds to an individual sample classified by PAM analysis (differentiated mast cell tumors are indicated in yellow, while undifferentiated ones in red). The value of each principal component is reported on the graph. The sum of the three principal components accounted to the 99.924% of the total variance.

The magnitude and pattern of gene expression of selected transcripts are represented in Figure 4. The heat map comparing the two groups displayed relatively consistent alterations in gene expression between the two categories. The statistical approach (PCA of all 51 MCT samples restricted to gene expression data of the 13 selected genes) confirmed this result. In details, the first three components, accounting for 99.924% of the total variance, identified two groups. The x-axis, explaining almost all the variance (99.746%), separated samples referable to the two identified categories (Figure 5).

### qPCR Analysis

The mRNA expression of the 13 target genes was finally evaluated by qPCR. A complete list of the thirteen selected target genes and the three ICGs (including sequences IDs used for primer design, primer pair sequences, probes and amplicon size) is presented in Table S5. Calibration curves revealed a PCR efficiency comprised in the range of acceptability (90–110%), an error value <0.2 and a dynamic range >9 cycles (except for centromere protein P (CENPP), characterized by a dynamic range of 6 cycles: Table S6). Table 3 showed the relative quantification values of each target gene in the 39 differentiated and 12 undifferentiated samples. Except for gelsolin (GSN), that was significantly down-regulated in undifferentiated group, all remaining target genes were significantly up-regulated (*p*<0.001) from about 2- up to 7-fold in undifferentiated MCTs. Spearman correlation analysis between FC obtained through DNA microarray and qPCR analysis was statistically significant (Spearman r = 0.88, *p*<0.001).

**Table 3:** Target genes mRNA expression in differentiated and undifferentiated MCT samples.

Target genes	qPCR results (RQ values ± SD)			
	Differentiated MCTs (n = 39)	Undifferentiated MCTs (n = 12)	*p* value	FC
CCNB2	0.25±0.18	0.97±0.28	*p*<0.001	3.88
CDC20	1.84±1.22	6.15±2.93	*p*<0.001	3.34
CDCA8	0.41±0.21	1.13±0.38	*p*<0.001	2.76
CENPP	0.58±0.24	1.09±0.34	*p*<0.001	1.88
FEN1	0.70±0.18	1.30±0.59	*p*<0.001	1.86
FOXM1	0.49±0.20	1.71±1.25	*p*<0.001	3.49
GSN	5.80±2.39	1.99±1.20	*p*<0.001	0.34
KPNA2	0.43±0.10	0.78±0.27	*p*<0.001	1.81
NUF2	0.39±0.23	2.76±2.57	*p*<0.001	7.08
NUSAP1	0.29±0.09	0.74±0.36	*p*<0.001	2.55
PRC1	0.43±0.28	2.05±1.11	*p*<0.001	4.77
RAD51	0.22±0.10	0.73±0.20	*p*<0.001	3.32
UBE2S	0.74±0.19	1.47±0.63	*p*<0.001	1.99

Data are expressed in arbitrary units. FC: -fold change.

### Survival Analysis

**Figure 6:**
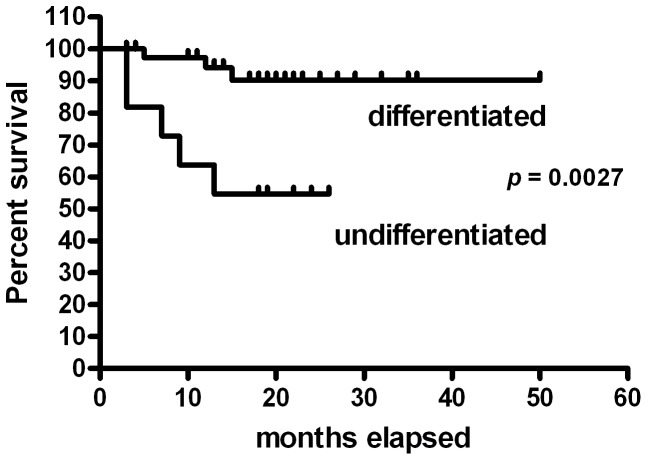
Survival curve of differentiated vs. undifferentiated mast cell tumors. Kaplan-Meyer survival plot stratified by molecular classification (differentiated and undifferentiated MCTs).

The molecular classification into differentiated and undifferentiated MCTs was considered as stratification variable for a survival time analysis (Figure 6). Differentiated MCTs showed a survival probability of 94% after 12 months, stabilizing at 90% after 15 months. Undifferentiated MCTs survival probability stabilized at 50% after 13 months. The survival curves of differentiated and undifferentiated samples were significantly different (*p* = 0.0026).

**Figure 7:**
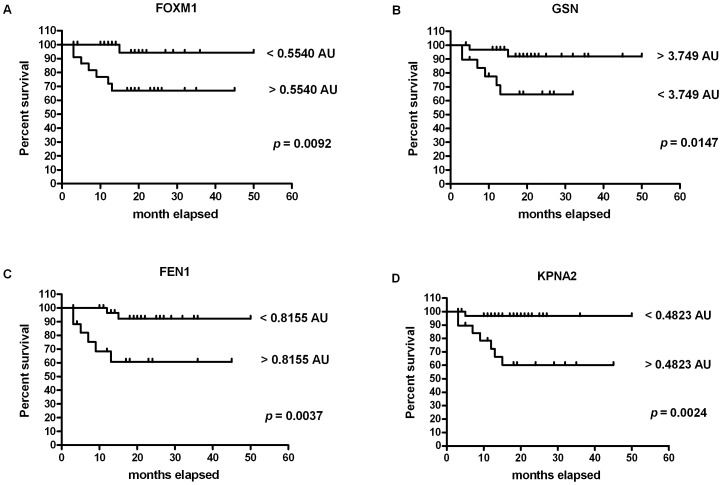
Survival curves for mortality due to mast cell tumor, stratified by marker gene mRNA expression cut point. (A) FOXM1. The cut-off chosen was 0.5540 AU (FOXM1<0.5540 AU: n = 28; FOXM1>0.5540 AU: n = 23). (B) GSN. The cut-off chosen was 3.749 AU (GSN >3.749: n = 31; GSN <3.749 AU: n = 20). (C) FEN1. The cut-off chosen was 0.8155 AU (FEN1<0.8155 AU: n = 33; FEN1>0.8155 AU: n = 18). (D) KPNA2. The cut-off chosen was 0.4823 AU (KPNA2<0.4823 AU: n = 31; KPNA2>0.4823 AU: n = 20).

To understand whether the target gene modulation was associated with an increased incidence of MCT-related mortality, ROC curve analysis was performed for each target. A significant association with MCT-related mortality was evidenced for Forkhead box M1 (FOXM1), GSN, Flap structure specific endonuclease 1 (FEN1) and Karyopherin alpha 2 (KPNA2). Specifically, the area under the ROC curve (AUC) for FOXM1 gene showed a value of 0.79 (95% CI: 0.60–0.98), and this one estimated the test global performance. By using the cut-off of 0.5540 arbitrary units (AU, RQ value), test sensitivity and the specificity were 87.5% (95% CI, 47.35% –99.68%) and 62.79% (95% CI, 46.72% –77.02%), respectively. Overall, 1 out of 28 cases with FOXM1<0.5540 AU and 7 out of 23 cases with FOXM1>0.5540 AU died because of MCT. With a RQ value <0.5540 AU the survival probability stabilized at 94% after 15 months, whereas for a RQ value >0.5540 AU the survival probability was 67% after 13 months. The two survival curves significantly differed (*p* = 0.0092: Figure 7A). About GSN, the best cut-off value was 3.749 AU (AUC: 0.74; 95% CI, 0.53–0.96), with a sensitivity of 75.0% (95% CI, 34.91%–96.81%) and a specificity of 67.44% (95% CI, 51.46%–80.92%). On the whole, 2 out of 31 cases with GSN >3.749 AU and 6 out of 20 cases with GSN <3.749 AU died of MCT. With a RQ value >3.749 AU the survival probability stabilized at 92% after 15 months, while with a RQ value <3.749 AU the survival probability was 65% after 13 months. The two survival curves were significantly different (*p* = 0.0147: Figure 7B). The AUC value for FEN1 was 0.72 (95% CI, 0.51–0.93). By using a cut-off of 0.8155 AU, the test sensitivity and specificity were 75.0% (95% CI, 34.91% –96.81%) and 69.77% (95% CI, 53.87% –82.82%), respectively. Overall, 2 out of 33 cases with FEN1<0.8155 AU and 6 out of 18 cases with FEN1>0.8155 AU died because of MCT. The survival probability with RQ <0.8155 was 96% after 12 months and stabilized at 92% after 15 months, whereas with RQ >0.8155 was 68% after 9 months and stabilized at 61% after 13 months. The two survival curves significantly differed (*p* = 0.0037: Figure 7C). Finally, the best identified cut-off value for KPNA2 was 0.4823 AU (AUC: 0.82; 95% CI, 0.65–0.99), with sensitivity and specificity of 87.5% (95% CI, 47.35%–99.68%) and 69.77% (95% CI, 53.87%–82.82%), respectively. Overall, 1 out of 31 cases with KPNA2<0.4823 AU and 7 out of 20 cases with KPNA2>0.4823 AU died because of MCT. With a RQ value <0.4823 the survival probability stabilized at 97% after 5 months, while with a RQ value >0.4823 the survival probability was 60% after 15 months. Even in this case, the two survival curves were significantly different (*p* = 0.0024: Figure 7D).

### qPCR Confirmatory Analysis

The potential use of a simplified gene expression profile that could be translated into a diagnostic platform to rapidly and accurately distinguish between differentiated and undifferentiated MCTs was finally evaluated. This step of validation consisted in the evaluation of the gene expression profiling of the 13 selected transcripts, by using a qPCR approach, in an independent cohort of 22 MCT samples. This step permitted to verify their utility to provide a molecular classification being independent from classical histological grading. Tumor samples were graded by pathologists who were unaware of the molecular results. Follow up data were also collected for each case.

**Figure 8:**
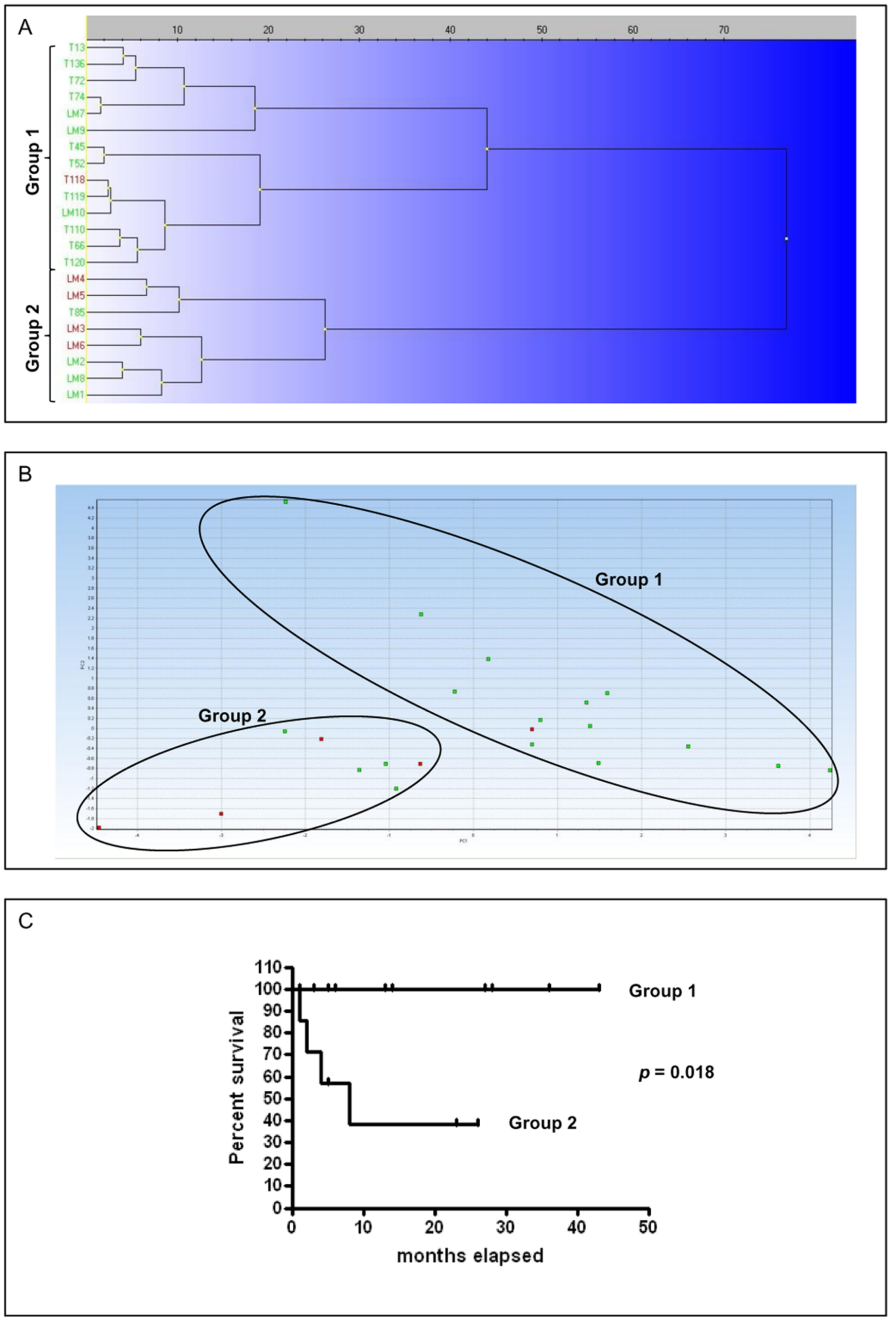
qPCR confirmatory analysis. Marker genes identified by class prediction analysis were amplified in an independent cohort of 22 mast cell tumors in order to comprehend their utility for mast cell tumor classification. Clustering analysis and PCA of gene expression data were performed using MultiD-Genex software for qPCR data, using the following settings: mean center scaling, Ward’s algorithm and Manhattan distance. (A) Clustering tree and (B) principal component analysis of independent mast cell tumor samples. Cases characterized by MCT-related death are indicated in red. The two groups identified are named group 1 and 2 (differentiated and undifferentiated MCTs, respectively). (C) Kaplan-Meyer survival plot stratified by molecular classification (group 1 and 2).

Data collected from qPCR analysis were analyzed by using the MultiD-Genex software. Clustering and PCA results are reported in Figure 8A–B. In PCA, the first two principal components accounted for 71.32% of the total variance. Both analyses showed the separation of samples into two main groups named Group 1 and 2, referable to differentiated and undifferentiated MCTs, respectively. Particularly, if we consider the histological grading, Group 1 (differentiated, n = 14) consisted of 7 Patnaik-G1 and G2 or 14 Kiupel L cases while Group 2 (undifferentiated, n = 8) consisted of 2 Patnaik-G1, 4 G2, 2 G3 or 5 Kiupel L and 3 H MCTs. By considering the outcome (dogs dead for MCT are indicated in red), in Group 2 50% of cases died for MCT, while in Group 1 only one case died during the follow up for MCT-unrelated causes; therefore it was not considered in the survival analysis (Figure 8C). The comparison of the two survival curves yielded a statistically significant difference (*p* = 0.0018); the survival probability of Group 2 stabilized at 38% after 5 months.

## Discussion

The transcriptome of canine mammary tumor, hemangiosarcoma, osteosarcoma, lymphoma, urinary bladder cancer, histiocytic sarcoma and melanoma has been recently characterized by using a DNA microarray approach [3], [4], [5], [6], [7], [8], [9], [10], [11], [12], [13], [14], [15].

To the best of authors’ knowledge, with the exception of a recent study comparing the proteome of 5 Kiupel L and 5 H MCTs [29], this is the first report focusing on the molecular characterization of canine MCT. Its importance in veterinary medicine is due not only to the high tumor frequency, but also to the highly variable biological behavior, often difficult to be predicted [16], [30].

In this scenario, we analyzed the transcriptome of 51 canine primary cutaneous MCTs aiming at identifying a gene signature of MCT aggressiveness and predicting *a priori* its biological behavior. The primary objective of this study was to provide a basis for the development of a simple diagnostic platform that could rapidly and accurately distinguish between differentiated and undifferentiated MCTs and support the histological grading, whose prognostic value is still questionable. Such a tool might be of great value to diagnose and treat MCT. Additionally, pet owners would greatly benefit from a more accurate survival time expectance when weighing their dog’s quality of life and their own monetary obligations in treatment decisions. Finally, the analysis of gene signature may allow the elucidation of either a single gene or a gene network that may be manipulated for treatment purposes.

To this aim, we performed preliminary HCL and PCA investigations by using the whole dataset (all informative genes) obtained by microarray analysis, but the extreme variability observed among the 51 samples tested did not permit to clearly separate samples into well-defined groups. Indeed, the sum of the first 2 principal components accounted for less than 30% of the total variance (data not shown). Thus, to define a reduced transcriptional profile permitting a better category assignment, we decided to use differentially expressed genes among the categories assigned by histological grading. Accordingly, the transcriptome was compared among Patnaik-G1, G2 and G3 MCTs and between Kiupel L and H MCTs by using SAM tool. In the first case, no differentially expressed genes were observed, due either to the high variability among samples or the imprecise/subjective grading. In the second case, a set of differentially expressed genes was observed, but the resulting PCA evidenced two partially overlapped categories (data not shown). The failure of these approaches prompted us to use an alternative approach, consisting in the choice of known reference samples to be used in class prediction analysis.

The choice of MCT cases to be used as known samples was based on histological criteria (2-tier histologic grading system and mitotic index) that permitted to obtain a 100% concordance among all pathologists. Statistical analysis of microarray data supported the choice and confirmed that reference samples belonged to two distinct categories.

Pathways analysis of DEGs revealed that cell cycle, DNA replication, nucleotide excision repair, p53 signaling pathway and pyrimidine metabolism were among the pathways deregulated in undifferentiated MCTs compared to well differentiated MCTs. In particular, our data highlights pathways that are important in rate of proliferation, malignant transformation, response to DNA damage and nucleotide metabolism. An altered expression of genes within these functional categories has been previously reported in the literature for other solid tumors, and these genes properly characterized the tumor when compared to its normal counterparts [3], [7]. In addition, the cell-cycle signature has been described to be useful to stratify canine and human osteosarcomas according to their biological behavior *in vivo* [10].

The class prediction analysis identified a set of 13 transcripts involved in malignant transformation that were able to accurately separate MCT samples into differentiated and undifferentiated ones. This panel of genes did not match with the proteins recently identified by using a proteomic approach. In this latter study, 5 Kiupel L and 5 H MCTs were compared, and in H MCTs a modulation of four stress response proteins (HSPA9, PDIA3, TCP1A and TCP1E) as well as of proteins mostly associated with cell motility and metastasis (WDR1, ACTR3, ANXA6, ANXA2, ACTB and transferrin) was observed [29]. Conversely, most genes here identified are coordinately regulated during mitosis [31], [32] and/or are part of the DNA damage checkpoint. Cell cycle pathway is in fact often deregulated in cancer during malignant transformation, and the increased DNA repair gene expression is most likely a consequence of increased DNA replication. As an example, pathways as cell cycle checkpoint, mitosis, spindle cell genes and DNA repair were found to be commonly up-regulated in canine mammary tumor and human breast cancer [5]. Most of the genes here identified for their over-expression in undifferentiated MCTs are involved in cell cycle and are described below.

Cyclin B2 (CCNB2) has a function at the transition from G2 to mitosis. Experimental evidence suggested that CCNB2 promoter activity is down-regulated by tumor suppressor p53 gene [33] and it may function as an oncogene [34]. An over-expression of this gene has been previously observed in human cervical cancer, invasive breast carcinoma, human astrocytoma, human pituitary adenoma, and canine osteosarcoma [31], [34], [35], [36], [10]; in addition, the relative expression of serum circulating CCNB2 mRNA in human cancer patients was significantly higher than in normal controls and benign disease group, and significantly correlated with cancer stage and metastasis status. Thus, it may have potential clinical applications in screening and monitoring of metastasis and therapeutic treatments [37].

The Forkhead box M1 (FOXM1) is an oncogenic transcription factor of the Forkhead family, whose expression is excluded in quiescent or differentiated cells, but highly elevated in proliferating and malignant cells [38]. FOXM1 is over-expressed in a variety of human tumors and plays a critical role in cancer development and progression [38].

Cell division cycle 20 homolog (CDC20) promotes spindle assembly checkpoint-mediated mitotic arrest through the mitotic checkpoint complex, induces apoptosis through degradation of anti-apoptotic proteins and triggers mitotic exit through CCNB degradation [39]. Recent studies have shown that CDC20 may function as an oncoprotein, promoting the development and progression of human cancers [40]. Results here obtained are in accordance with human literature, where an up-regulation of this gene was observed in cervical cancer, primary non-small cell lung cancer, pancreatic ductal adenocarcinoma, urothelial bladder cancer and colorectal cancer, too [31], [41], [42], [43], [44]; therefore, it may serve as a potential prognostic biomarker, as already described in human oncology.

Cell division cycle associated protein 8 (CDCA8) is a component of the chromosomal passenger complex (CPC), a complex that acts as a key regulator of mitosis. The CPC complex has essential functions at the centromere in ensuring correct chromosome alignment and segregation and is required for chromatin-induced microtubule stabilization and spindle assembly. It exhibited frequent and robust up-regulation in cervical cancer and chemoresistant ovarian cancer [45], [46].

NUF2 (NDC80 kinetochore complex component, homolog) or CDCA1 (cell division cycle associated protein 1) is another component of the essential kinetochore-associated NDC80 complex, which is required for chromosome segregation and spindle checkpoint activity during mitosis. An over-expression of this transcript has been previously observed in colorectal and gastric cancers, as well as in serous adenocarcinomas [47], [48]. The silencing of this gene through RNA interference leads to increased apoptosis [48], and suggests that its expressional control could be utilized for molecular target therapy in patients affected by colorectal and gastric carcinoma [47].

Nucleolar and spindle-associated protein 1 (NUSAP1) plays a role in spindle microtubule organization. An over-expression of this gene has been reported in cervical cancer, grade III versus grade I meningioma, in recurrent prostate cancer after radical prostatectomy and breast cancer [31], [49], [50], [51]. It has been also suggested as an early molecular marker of ductal carcinoma in situ and invasive carcinoma [51] and as a prognostic factor in metastatic melanoma where it is negatively associated with survival [52].

Protein Regulator of Cytokinesis 1 (PRC1) is present at high levels during the S and G2/M phases of mitosis but its levels drop dramatically when the cell exits mitosis and enters the G1 phase. Further than its role in human cervical cancer [31], it has been recognized as a therapeutic gene for its cancer-specific over-expression in breast cancer cell lines and patient tissues [53].

Centromere protein P (CENPP) is a novel centromere protein that has an important role during interphase of cell cycle and it is required for proper kinetochore function and mitotic progression [55]. An overexpression of centromere proteins has been observed in various types of human cancers and was significantly correlated with tumor grade and survival [54], [55], [56], [57], [58].

Ubiquitin-conjugating enzyme E2S (UBE2S) gene encodes a member of the ubiquitin-conjugating enzyme family that works with the anaphase-promoting complex ubiquitin ligase ubiquitylating protein substrates whose degradation regulates progress through mitosis [59]. In a recent work in breast cancer [60] authors identified, through weighted gene co-expression network analysis, a cluster of proliferation-related genes including UBE2S that, when up-regulated, were correlated to increased tumor grade and were associated with poor survival.

The last gene belonging to mitosis pathway, namely GSN, was the only gene down-regulated in undifferentiated MCTs. It has been reported to be an onco-suppressor participating in the regulation of the apoptotic process and interacting with p53 [61]. Its over-expression causes cell cycle arrest or delay at the G2/M phase of the cell cycle and inhibition of tumor growth, as demonstrated in an orthotopic bladder cancer nude mouse model [62]. GSN is down-regulated in human breast cancer tissues compared to controls and its transcript level is linked with metastasis development and death [63]. Furthermore, GSN has been proposed as serum biomarker and potential target for gene therapy in human osteosarcoma [64].

Other two potential markers, Flap endonuclease 1 (FEN1) and RAD51 are involved in DNA repair pathway. FEN1 is a structure-specific endonuclease best known for its critical roles in Okazaki fragment maturation, DNA repair and apoptosis-induced DNA fragmentation [65]. It plays an essential role in maintaining genomic stability and preventing tumorigenesis, thus acting as a tumor suppressor [66]. FEN1 is significantly up-regulated in multiple human cancers and its aberrant expression in tumor cells is associated with hypomethylation of the CpG islands within the FEN1 promoter [67].

RAD51 protein plays a key role in homologous recombination. It has been shown to be up-regulated in many human cancers, especially higher grade, chemoresistant and radioresistant tumors [68]. Expression is tightly regulated in normal cells, with dysregulation leading to genomic instability and possibly contributing to oncogenesis [68]. RAD51 mRNA amount was increased in laser-microdissected mammary simple carcinomas when compared to adenomas or non neoplastic mammary gland of the same dog, indicating a genomic instability in RAD51-expressing cells in carcinomas [69], [70].

Finally, Karyopherin α 2 (KPNA2) has recently emerged as a potential biomarker in multiple human cancer forms [71]. Owing to its role in nucleocytoplasmic transport, where it mediates the translocation of a multitude of proteins, KPNA2 is involved in many cellular processes [72]. The aberrant high levels observed in human cancer tissue (i.e., gastric adenocarcinoma and epithelial ovarian cancer) have been associated with poor prognosis, prompting the idea that KPNA2 plays a role in carcinogenesis [73], [74]. Studies in cancer cells demonstrated that KPNA2 deregulation affects malignant transformation, thus it was considered a potentially relevant therapeutic target [72].

Molecular pathology, a scientific approach defining cancer subtypes based on the underlying molecular footprints, has led to the discovery of subtypes in several different tumors. Nevertheless, it has been demonstrated that this approach works at its best when cancer subtypes based on genetic profiles are already known: whenever the different subtypes of the same cancer and the number of patients belonging to each of them are known, then a statistical model could be built to associate a specific gene expression profiling, typical of a subtype of cancer, to an individual patient [75]. In the present study, the approach of using well known histological categories did not produce useful results; thus, an alternative approach consisting of a predictive analysis was used. Alternative statistical methods to identify subtypes exist, but they can generate classifications that lack clinical relevance [75]. Nevertheless, in the present work we were able to separate MCTs in two molecular subtypes that did not perfectly match with Patnaik-G1, G2, G3 or Kiupel L and H grades, defined by conventional histological grading. Conversely, they referred to differentiated and undifferentiated MCTs, whose prognosis was largely documented in the literature [76] and here confirmed by survival analysis.

Meanwhile, presented results confirmed the usefulness of a transcriptomic approach in the definition of a signature that could segregate molecular subtypes of the same tumor with different survival probabilities [3], [10], [77]. Thus, the molecular classification coupled with the histological grading, universally recognized as a reference for MCT prognosis prediction but still presenting some gaps (i.e., Patnaik-G2 MCT), might permit a better characterization of the biologic behavior of the tumor.

As genome-wide gene expression profiling is cost-prohibitive and impractical, we selected 13 simple qPCR assays to stratify MCTs into differentiated and undifferentiated cases. The reliability of the gene set was tested, by means of a qPCR approach, upon a separate cohort of MCT samples for which a complete follow up was available. Even in this case, we observed a statistical significant association between molecular classification and survival time, confirming the potential usefulness of these results to develop a rapid and relatively cheap benchtop diagnostic test based on the expression of 13 genes that can classify canine MCTs into one of these 2 subgroups, and enabling a direct clinical application of our results. Worth mentioning, in veterinary medicine a benchtop diagnostic test composed of 4 genes has been previously proposed for canine lymphoma [11].

As a whole, this study allowed us to fill a gap in the tumor understanding and it contributed to the uncertainty about the relevance and utility of morphological classification systems. In addition, the transcripts here identified not only may predict prognosis (relative quantification values were significantly associated with poor survival), but may also be selected as future targets for therapy (gene therapy or chemotherapy), as previously reported in various human cancers [37], [47], [53], [64], [72].

In the future, a study population with a larger sample set of tumors might provide a more robust basis to confirm and refine segregation between differentiated and undifferentiated MCTs.

## Conclusions

In conclusion, the transcriptome of canine cutaneous MCT was characterized for the first time by using a DNA microarray approach. Data obtained allowed the identification of a signature of aggressiveness in canine MCT; particularly, a set of 13 potential biomarkers identified through a class prediction analysis segregated MCT samples into two distinct molecular categories, namely differentiated and undifferentiated MCTs. The molecular classification into differentiated and undifferentiated MCTs as well as the mRNA expression of FOXM1, GSN, FEN1 and KPNA2 were significantly associated with survival. The prognostic value of the molecular classification was also confirmed in a separate cohort of MCT cases, for which an accurate follow up was available. Thus, this set of genes could be useful to develop a benchtop diagnostic test that could support canine MCT histological grading and consequently having prognostic relevance. Finally, analysis of gene signature allowed elucidation of single genes or genetic pathways (mitosis and DNA repair), which could be considered for future diagnostic and treatment purposes as already performed in human oncology.

## Supporting Information

Table S1 Demographic characteristics of complete cohort of 73 dogs with cutaneous mast cell tumor and restricted groups (n = 51 and n = 22) analyzed by gene expression profiling (GEP cohort) and quantitative Real Time PCR confirmatory analysis (dogs not on array), respectively. The table describes the characteristics (sex, age, breed, histological classification and survival data) of the 73 dogs selected for the study, including the 2 population subsets (gene expression profiling cohort and dogs not on array).
(DOCX)

Table S2 Histological grading and mitotic index of reference samples (differentiated and undifferentiated) used in the class prediction analysis. The table describes the list of reference differentiated and undifferentiated mast cell tumor samples used for class prediction analysis and the corresponding Patnaik and Kiupel histological grading and mitotic index.
(DOCX)

Table S3 Down-regulated genes (n = 147) in undifferentiated reference samples and corresponding –fold changes (FC). The table describes the list of the entire set of down-regulated genes (n = 147) obtained through the comparison of differentiated and undifferentiated reference samples transcriptome. The –fold change for each probe is also reported.
(DOCX)

Table S4 Up-regulated genes (n = 450) in undifferentiated reference samples and corresponding –fold changes (FC). The table describes the list of the entire set of up-regulated genes (n = 450), obtained through the comparison of differentiated and undifferentiated reference samples transcriptome. The –fold change for each probe is also reported.
(DOCX)

Table S5 List of selected genes: Ensembl Genome Browser sequence ID, primer sequences, UPL probe and amplicon size. The table defines target and internal control genes selected for qPCR analysis and all the information about qPCR assay design: transcript Ensembl Genome Browser ID, primer sequences, UPL probe and amplicon size.
(DOCX)

Table S6 qPCR assay parameters: primer concentration, efficiency, error value and dynamic range. The table defines the main features of qPCR assays obtained during their setting up. In particular primer concentration and efficiency, error value and dynamic range are reported.
(DOCX)
