# Supplementary material for: Global Gene Expression Analysis of Canine Cutaneous Mast Cell Tumor: Could Molecular Profiling Be Useful for Subtype Classification and Prognostication?
Source: PLoS One. 2014 Apr 18;9(4):e95481. doi: 10.1371/journal.pone.0095481 (PMC3991658; doi:10.1371/journal.pone.0095481)
Supplement: Table S1 — Demographic characteristics of complete cohort of 73 dogs with cutaneous mast cell tumor and restricted groups (n = 51 and n = 22) analyzed by gene expression profiling (GEP cohort) and quantitative Real Time PCR confirmatory analysis (dogs not on array), respectively. The table describes the characteristics (sex, age, breed, histological classification and survival data) of the 73 dogs selected for the study, including the 2 population subsets (gene expression profiling cohort and dogs not on array). (DOCX) [file pone.0095481.s001.docx]

**Table S1.** Demographic characteristics of complete cohort of 73 dogs with cutaneous mast cell tumor and restricted groups (n = 51 and n = 22) analyzed by gene expression profiling (GEP cohort) and quantitative Real Time PCR confirmatory analysis (dogs not on array), respectively.

|  | Complete cohort  (n = 73)  Number (%) | GEP cohort  (n = 51)  Number (%) | Dogs not on array  (n = 22)  Number (%) |
| --- | --- | --- | --- |
| **SEX** |  |  |  |
| Male | 24 (32.9) | 19 (37.3) | 5 (22.7) |
| Female | 46 (63.0) | 30 (58.8) | 16 (72.7) |
| Data not available | 3 (4.1) | 2 (3.9) | 1 (4.6) |
| **AGE** |  |  |  |
| Mean age at diagnosis (yy) ± SD | 8.1 ± 2.8 | 8.6 ± 2.7 | 7.0 ± 2.6 |
| Range (yy) | 2 - 14 | 3 - 14 | 2 - 12 |
| Data not available | 2 (2.7) | 1 (2.0) | 1 (4.6) |
| **BREED** |  |  |  |
| Pure breed* | 49 (67.1) | 36 (70.6) | 13 (59.1) |
| Cross-bred | 23 (31.5) | 14 (27.5) | 9 (40.9) |
| Data not available | 1 (1.4) | 1 (1.9) | 0 (0.0) |
| **HISTOLOGICAL CLASSIFICATION** |  |  |  |
| Patnaik | G1 (32, 43.8)  G2 (35, 48.0)  G3 (6, 8.2) | G1 (23, 45.1)  G2 (24, 47.1)  G3 (4, 7.8) | G1 (9, 40.9)  G2 (11, 50.0)  G3 (2, 9.1) |
| Kiupel | L (63, 86.3)  H (10, 13.7) | L (44, 86.3)  H (7, 13.7) | L (19, 86.4)  H (3, 13.6) |
| **SURVIVAL DATA** |  |  |  |
| Median survival time (months) | 17 | 18 | 13 |
| Alive | 50 (68.5) | 34 (66.6) | 16 (72.7) |
| Dead for mast cell tumor | 13 (17.8) | 8 (15.7) | 5 (22.7) |
| Dead for MCT unrelated causes | 6 (8.2) | 6 (11.8) | 0 (0.00) |
| Outcome lost | 4 (5.5) | 3 (5.9) | 1 (4.6) |

* Labrador Retriever (15), Boxer (11), Golden Retriever (4), English Setter (2), Dachshund (2), Cocker Spaniel (2), Dogo Argentino (2), American Staffordshire Terrier (1), Boston Terrier (1), Bouvier des Flandres (1), Cane Corso (1), Chihuahua (1), Dalmatian (1), Dogue de Bordeaux (1), Pit bull (1), Shi Tzu (1), Shar-pei (1), and Rottweiler (1).
