# Supplementary material for: Global Gene Expression Analysis of Canine Cutaneous Mast Cell Tumor: Could Molecular Profiling Be Useful for Subtype Classification and Prognostication?
Source: PLoS One. 2014 Apr 18;9(4):e95481. doi: 10.1371/journal.pone.0095481 (PMC3991658; doi:10.1371/journal.pone.0095481)
Supplement: Table S2 — Histological grading and mitotic index of reference samples (differentiated and undifferentiated) used in the class prediction analysis. The table describes the list of reference differentiated and undifferentiated mast cell tumor samples used for class prediction analysis and the corresponding Patnaik and Kiupel histological grading and mitotic index. (DOCX) [file pone.0095481.s002.docx]

**Table S2.** Histological grading and mitotic index of reference samples (differentiated and undifferentiated) used in the class prediction analysis.

| **DIFFERENTIATED SAMPLES** | | | |
| --- | --- | --- | --- |
| ID sample | Patnaik  Histological classification | Kiupel  Histological classification | Mitotic  index |
| T48 | G2 | L | 0 |
| T50 | G1 | L | 0 |
| T55 | G2 | L | 0 |
| T62 | G1 | L | 0 |
| T64 | G2 | L | 0 |
| T84 | G2 | L | 1 |
| T88 | G1 | L | 0 |
| T92 | G1 | L | 0 |
| T100 | G2 | L | 0 |
| T101 | G1 | L | 0 |
| T112 | G1 | L | 0 |
| T130 | G2 | L | 3 |
| T131 | G2 | L | 5 |
| **UNDIFFERENTIATED SAMPLES** | | | |
| T9 | G2 | H | 10 |
| T57 | G2 | H | 8 |
| T73 | G3 | H | 15 |
| T90 | G3 | H | 45 |
| ARINI | G3 | H | > 7 |
