# Supplementary material for: Global Gene Expression Analysis of Canine Cutaneous Mast Cell Tumor: Could Molecular Profiling Be Useful for Subtype Classification and Prognostication?
Source: PLoS One. 2014 Apr 18;9(4):e95481. doi: 10.1371/journal.pone.0095481 (PMC3991658; doi:10.1371/journal.pone.0095481)
Supplement: Table S3 — Down-regulated genes (n = 147) in undifferentiated reference samples and corresponding –fold changes (FC). The table describes the list of the entire set of down-regulated genes (n = 147) obtained through the comparison of differentiated and undifferentiated reference samples transcriptome. The –fold change for each probe is also reported. (DOCX) [file pone.0095481.s003.docx]

**Table S3.** Down-regulated genes (n = 147) in undifferentiated reference samples and corresponding –fold changes (FC).

| **DOWN-REGULATED GENES (n = 147)** | | | | | | | | | |
| --- | --- | --- | --- | --- | --- | --- | --- | --- | --- |
| **Transcript** | **FC** | **Transcript** | **FC** | **Transcript** | **FC** | **Transcript** | **FC** | **Transcript** | **FC** |
| TC53524 | 0.247 | A_11_P112391 | 0.400 | RIN2 | 0.428 | JAM2 | 0.296 | TC74122 | 0.172 |
| GSN | 0.342 | CNRIP1 | 0.177 | DN372747 | 0.423 | CO635051 | 0.360 | TC49690 | 0.263 |
| DN392973 | 0.483 | TC64189 | 0.354 | DN429088 | 0.363 | CF410229 | 0.432 | JAZF1 | 0.310 |
| CD99 | 0.292 | SNX33 | 0.326 | CO686371 | 0.433 | CRIP1 | 0.399 | CRIM1 | 0.441 |
| TC68428 | 0.348 | C1S | 0.199 | DN434984 | 0.341 | CO714279 | 0.368 | ADAMTSL3 | 0.341 |
| TC58832 | 0.208 | SEPP1 | 0.128 | CAMK1D | 0.476 | JAM2 | 0.374 | FRMD3 | 0.251 |
| IL34 | 0.323 | LOC612378 | 0.246 | TC76052 | 0.315 | TC78163 | 0.472 | TC60391 | 0.369 |
| ENSCAFT00000002285 | 0.395 | DDB2 | 0.467 | LOC484172 | 0.334 | ARSA | 0.475 | TWIST | 0.370 |
| JAZF1 | 0.202 | JAZF1 | 0.261 | TC58991 | 0.476 | TC52084 | 0.397 | PEPD | 0.319 |
| TC61190 | 0.478 | ABLIM1 | 0.428 | DN378832 | 0.274 | TC70360 | 0.276 | ABHD14B | 0.437 |
| GSN | 0.214 | LOC478170 | 0.283 | TC76786 | 0.290 | MAML2 | 0.423 | DN873785 | 0.185 |
| DN875961 | 0.297 | LIPA | 0.363 | CX011584 | 0.249 | LDHD | 0.431 | MAPK8IP1 | 0.420 |
| TC68256 | 0.373 | TC57014 | 0.309 | TC56539 | 0.227 | DQ195096 | 0.422 | C1S | 0.193 |
| CALHM2 | 0.400 | A_11_P064056 | 0.228 | TC52265 | 0.307 | GDA | 0.160 | DN379483 | 0.156 |
| LOC476059 | 0.364 | DN372734 | 0.141 | MXRA5 | 0.362 | CF410772 | 0.196 | TC67881 | 0.251 |
| GSN | 0.193 | LOC611318 | 0.321 | TC49663 | 0.129 | LOC475615 | 0.392 | BU744762 | 0.342 |
| TC51073 | 0.469 | JAM2 | 0.273 | TC61253 | 0.282 | TC60490 | 0.453 | BAIAP2 | 0.424 |
| A_11_P097126 | 0.454 | JAM2 | 0.276 | MYO1E | 0.466 | TC62629 | 0.457 | TC50470 | 0.223 |
| LOC476059 | 0.348 | JAM2 | 0.287 | ABLIM1 | 0.454 | TC57285 | 0.170 | TSPAN13 | 0.367 |
| SEPP1 | 0.167 | SNX9 | 0.464 | LOC480601 | 0.488 | TC63276 | 0.289 | SBF2 | 0.458 |
| CALHM2 | 0.437 | A_11_P092121 | 0.377 | TGFBR3 | 0.286 | MYO1E | 0.496 | DN747503 | 0.233 |
| TSPAN13 | 0.341 | TC53007 | 0.430 | ALDH3B1 | 0.383 | TC62226 | 0.051 | SERPINB8 | 0.416 |
| LOC489163 | 0.248 | TXNIP | 0.437 | C3 | 0.170 | TC47656 | 0.326 | TIMP3 | 0.185 |
| LOC611318 | 0.442 | ABLIM1 | 0.437 | TXNIP | 0.447 | TC68529 | 0.297 | ITIH5 | 0.231 |
| GHR | 0.226 | TC54889 | 0.369 | ADD3 | 0.465 | TC69012 | 0.366 | CO630369 | 0.416 |
| LOC607548 | 0.468 | LIPA | 0.363 | TC73621 | 0.471 | TC50186 | 0.443 | BLNK | 0.441 |
| CDON | 0.149 | TXNIP | 0.406 | ABLIM1 | 0.450 | TC68116 | 0.427 | CO678475 | 0.296 |
| ARRB1 | 0.417 | OLFML1 | 0.284 | LOC484172 | 0.307 | ZNF385D | 0.302 |  |  |
| SEPP1 | 0.181 | DN373009 | 0.432 | FYCO1 | 0.467 | TC58698 | 0.410 |  |  |
| CF409846 | 0.356 | DN747772 | 0.408 | TC52515 | 0.454 | CRIM1 | 0.439 |  |  |
