# Supplementary material for: Global Gene Expression Analysis of Canine Cutaneous Mast Cell Tumor: Could Molecular Profiling Be Useful for Subtype Classification and Prognostication?
Source: PLoS One. 2014 Apr 18;9(4):e95481. doi: 10.1371/journal.pone.0095481 (PMC3991658; doi:10.1371/journal.pone.0095481)
Supplement: Table S4 — Up-regulated genes (n = 450) in undifferentiated reference samples and corresponding –fold changes (FC). The table describes the list of the entire set of up-regulated genes (n = 450), obtained through the comparison of differentiated and undifferentiated reference samples transcriptome. The –fold change for each probe is also reported. (DOCX) [file pone.0095481.s004.docx]

**Table S4.** Up-regulated genes (n = 450) in undifferentiated reference samples and corresponding –fold changes (FC).

| **UP-REGULATED GENES (n = 450)** | | | | | | | | | |
| --- | --- | --- | --- | --- | --- | --- | --- | --- | --- |
| **Transcript** | **FC** | **Transcript** | **FC** | **Transcript** | **FC** | **Transcript** | **FC** | **Transcript** | **FC** |
| RRM2 | 9.144 | TC53706 | 3.336 | DIAPH3 | 4.855 | MPP4 | 13.290 | LOC483857 | 2.073 |
| TC54076 | 2.987 | MAD2L1 | 4.051 | UCHL1 | 23.412 | TC57198 | 2.705 | TC76074 | 3.629 |
| UBE2S | 3.032 | PLK1 | 3.208 | WRB | 2.225 | DEPDC1 | 19.388 | SDSL | 5.273 |
| CDC20 | 7.260 | TC57735 | 6.013 | BU746162 | 8.426 | LOC612464 | 9.756 | TC72238 | 3.781 |
| CX988067 | 2.935 | TC47358 | 7.507 | E2F8 | 5.038 | CBX5 | 2.480 | FABP6 | 2.621 |
| CENPP | 3.395 | FOXM1 | 6.711 | UBE2T | 5.277 | TC61740 | 2.845 | LIN9 | 2.115 |
| TPX2 | 4.708 | CASC5 | 7.236 | ACSL4 | 3.369 | TK1 | 3.473 | TC56112 | 2.872 |
| PRC1 | 5.938 | TOP2A | 8.697 | RAD18 | 2.055 | DN745126 | 2.550 | TC58344 | 2.025 |
| PRC1 | 6.018 | POLA2 | 2.275 | TC50933 | 2.133 | SLC25A33 | 2.139 | GUCA1B | 2.059 |
| DN409432 | 7.584 | CO694371 | 2.844 | TC63896 | 2.385 | MSI2 | 2.562 | TC77196 | 2.353 |
| SHCBP1 | 12.059 | TC75162 | 3.997 | CDC6 | 9.298 | ESPL1 | 4.074 | TC58088 | 4.521 |
| NUF2 | 8.454 | ESCO2 | 8.068 | PPID | 2.120 | DR104974 | 3.475 | STAT4 | 2.008 |
| FOXM1 | 7.082 | TC60941 | 2.575 | LOC609400 | 6.250 | PTTG1 | 3.231 | TC64424 | 2.788 |
| AURKA | 3.501 | ECT2 | 6.992 | LOC610710 | 8.661 | APOA2 | 4.675 | CTRL | 2.089 |
| DCK | 4.889 | SPATA5L1 | 2.051 | A_11_P106436 | 3.135 | A_11_P0000026198 | 3.104 | MCM7 | 2.481 |
| A_11_P067791 | 7.933 | A_11_P085411 | 5.441 | LOC487996 | 2.388 | TC59682 | 3.433 | UBE2A | 2.049 |
| SHCBP1 | 9.297 | GMNN | 2.031 | MXD3 | 2.033 | LOC611930 | 7.821 | TC76149 | 4.414 |
| AURKA | 3.907 | CDCA2 | 8.577 | STK39 | 2.838 | A_11_P071466 | 4.917 | GTF2IRD1 | 2.715 |
| CEL | 5.666 | NDC80 | 6.201 | MMD | 5.289 | DR105840 | 2.125 | LOC611752 | 2.826 |
| A_11_P074386 | 5.003 | MCM5 | 4.070 | DT540165 | 2.013 | OTOR | 3.622 | TC55821 | 2.560 |
| TPX2 | 4.334 | CDCA2 | 8.022 | SRGN | 2.236 | PSMG3 | 2.101 | ERAS | 3.214 |
| CCNB2 | 7.420 | DN749185 | 6.626 | CBX5 | 2.241 | A_11_P092881 | 2.782 | STK38L | 2.689 |
| DN746594 | 2.944 | CENPH | 4.558 | TC61252 | 2.128 | BM537446 | 2.895 | LOC479235 | 2.344 |
| RAD51 | 5.790 | CDC6 | 8.382 | CKAP2 | 2.652 | A_11_P0000028889 | 4.624 | CTRL | 2.146 |
| TC50815 | 3.607 | MTHFD2 | 2.340 | PLSCR1 | 6.931 | TC50602 | 2.289 | TC47147 | 3.976 |
| GSG2 | 4.914 | CDCA2 | 6.365 | A_11_P218943 | 3.271 | LOC480351 | 2.452 | NRG1 | 3.972 |
| CDC45L | 8.031 | A_11_P108026 | 5.110 | TC57653 | 4.615 | CF409927 | 3.980 | CTRL | 2.084 |
| DN745205 | 5.013 | TC62025 | 4.105 | RAD23A | 2.266 | OBFC2A | 2.233 | TMC6 | 2.588 |
| TC46778 | 5.883 | LOC488305 | 3.045 | LOC608641 | 5.627 | A_11_P0000029638 | 3.105 | CN002515 | 4.286 |
| LOC478258 | 7.042 | GMNN | 2.044 | ORC1L | 3.633 | TC58160 | 2.222 | A_11_P0000033212 | 4.333 |
| NUF2 | 8.162 | A_11_P0000026218 | 3.182 | DN744087 | 2.247 | KIF22 | 2.039 | TC65510 | 2.648 |
| FEN1 | 2.056 | LOC609701 | 8.298 | MAP4K2 | 2.048 | DN272305 | 2.179 | DN745459 | 5.500 |
| LOC480469 | 3.076 | NEK2 | 6.135 | TC54838 | 3.592 | FANCD2 | 2.274 | STK39 | 2.075 |
| CCNB2 | 6.046 | NEK2 | 5.101 | A_11_P115026 | 2.366 | RAD23A | 2.933 | TC76795 | 3.824 |
| UBE2S | 3.101 | A_11_P082291 | 4.673 | TC47516 | 5.264 | SRGN | 3.064 | A_11_P0000033048 | 2.225 |
| CCNB2 | 6.632 | ENO3 | 2.325 | CO590715 | 2.532 | LOC607729 | 2.184 | TC74097 | 2.160 |
| TTK | 8.041 | AURKB | 2.193 | TC57241 | 9.989 | LOC608611 | 2.148 | TC56442 | 5.425 |
| TC60372 | 3.471 | CEP76 | 2.406 | TC51601 | 2.079 | CO704124 | 2.215 | LOC610643 | 2.417 |
| ANLN | 6.368 | TC47515 | 9.157 | SAG | 8.320 | NCAPG | 5.321 | CDCA3 | 2.086 |
| UHRF1 | 3.505 | WHSC1 | 3.359 | MCM3 | 2.750 | A_11_P0000030681 | 2.506 | HSPA4L | 2.871 |
| ENSCAFT00000020009 | 4.391 | TC47515 | 9.051 | BTBD14B | 2.264 | NCAPG2 | 2.981 | LOC611180 | 4.372 |
| GALE | 2.624 | A_11_P102556 | 6.072 | A_11_P074356 | 3.358 | TC70211 | 2.638 | A_11_P098706 | 2.374 |
| BU749178 | 2.357 | PLK4 | 7.912 | TC59936 | 2.260 | CARHSP1 | 2.512 | ENSCAFT00000020009 | 7.056 |
| INCENP | 2.025 | POLE | 3.895 | TC53889 | 6.480 | TROAP | 2.217 | CO707800 | 2.019 |
| TC62240 | 4.919 | A_11_P0000031071 | 7.543 | MCM4 | 2.327 | A_11_P092986 | 2.014 | NFE2 | 2.618 |
| CX987798 | 4.355 | A_11_P084621 | 3.900 | BU745610 | 2.191 | CENPI | 4.832 | DR104974 | 2.705 |
| LOC609400 | 6.398 | ENO3 | 2.370 | BRCA1 | 4.020 | LIN9 | 2.143 | TACC3 | 3.609 |
| TC72941 | 8.426 | A_11_P0000033444 | 8.601 | MCM6 | 2.349 | LOC489765 | 2.326 | LOC491080 | 2.014 |
| CDCA3 | 7.149 | VRK1 | 2.191 | KIF11 | 8.959 | PDIA6 | 2.059 | TC53148 | 4.909 |
| TK1 | 3.255 | TC70525 | 3.241 | CCNB3 | 10.734 | A_11_P0000026629 | 4.406 | A_11_P072976 | 5.479 |
| A_11_P056666 | 3.461 | CCNE1 | 3.422 | A_11_P111321 | 5.999 | LOC478493 | 2.980 | A_11_P099831 | 2.082 |
| DN331815 | 4.448 | A_11_P0000028696 | 3.720 | APOA2 | 2.243 | A_11_P079076 | 5.093 | CO693913 | 2.465 |
| DN331815 | 6.005 | MMD | 5.951 | SAAL1 | 2.103 | SLC25A33 | 2.174 | DEFB3L | 6.902 |
| CKAP2 | 2.579 | KIF2C | 3.349 | KIF15 | 4.271 | DN878400 | 2.564 | LOC611419 | 2.508 |
| DEPDC1B | 6.825 | KIF18A | 2.968 | A_11_P0000034257 | 4.559 | HMGB2 | 2.675 | LOC481790 | 2.729 |
| BUB1B | 6.652 | TC66404 | 3.947 | FANCM | 5.802 | RSPH10B | 3.110 | IL5 | 4.747 |
| KIF4A | 4.463 | LOC491373 | 3.765 | TC76832 | 2.855 | YTHDF3 | 2.914 | TC59006 | 2.152 |
| TC57491 | 3.590 | LOC479209 | 2.764 | TC64335 | 5.139 | XPOT | 2.029 | UGT2A1 | 7.193 |
| TC74516 | 2.945 | CEP76 | 2.200 | DR106666 | 2.272 | TSGA14 | 2.145 | HPDL | 2.073 |
| CCNA2 | 8.801 | MAPK6 | 2.215 | LOC612032 | 3.580 | DDC | 4.442 | A_11_P107916 | 3.098 |
| A_11_P0000032690 | 7.787 | TK1 | 3.397 | CN004173 | 2.257 | TC62584 | 3.548 | MCM7 | 2.535 |
| DR103312 | 7.362 | PBK | 7.661 | DT536829 | 12.446 | LOC609286 | 2.040 | SERPINB4 | 8.666 |
| CBFB | 2.456 | HMGB3 | 3.964 | CARHSP1 | 3.286 | A_11_P084601 | 2.044 | E2F1 | 2.082 |
| SYNCRIP | 2.132 | MCM6 | 2.206 | LOC488268 | 8.071 | CLCN1 | 8.063 | DN876136 | 2.090 |
| A_11_P094866 | 3.947 | A_11_P084616 | 3.683 | ENSCAFT00000036918 | 2.996 | A_11_P0000023542 | 2.357 | PCDHGA11 | 2.234 |
| DR103312 | 5.866 | TIAM2 | 2.280 | RFC4 | 2.832 | CASD1 | 2.101 | DN754464 | 2.483 |
| PKMYT1 | 2.592 | A_11_P086406 | 5.027 | LOC490715 | 2.376 | TC54479 | 5.181 | CCNB1IP1 | 2.594 |
| NCAPD2 | 2.368 | LOC477773 | 3.370 | LOC490715 | 2.512 | CF409988 | 2.984 | ME2 | 2.088 |
| DLGAP5 | 12.039 | ANLN | 5.058 | TC58554 | 3.226 | ATAD2 | 2.414 | TC64050 | 2.593 |
| A_11_P101571 | 4.052 | A_11_P099056 | 4.502 | LOC486404 | 4.985 | TC53570 | 2.454 | HIF1A | 2.258 |
| TC57939 | 2.490 | SPC25 | 12.428 | A_11_P058936 | 2.246 | A_11_P0000031692 | 4.782 | DR104796 | 25.343 |
| LOC609701 | 5.973 | MCM10 | 7.013 | TC46593 | 3.206 | PDIA6 | 2.009 | A_11_P082221 | 4.168 |
| CENPA | 5.351 | KIF15 | 5.412 | TC67246 | 3.217 | A_11_P055476 | 3.102 | A_11_P0000027313 | 2.299 |
| CHAF1B | 2.522 | TC73933 | 4.221 | TC49021 | 4.954 | DDC | 5.155 | TC76999 | 2.266 |
| RACGAP1 | 5.704 | RHOH | 3.267 | TC52679 | 2.987 | SLC6A6 | 2.655 | CENPQ | 3.179 |
| LOC480469 | 3.367 | DN439577 | 4.004 | LOC489903 | 2.469 | MRPS6 | 2.413 | CDT1 | 2.006 |
| NEK2 | 6.995 | NUP210L | 4.255 | GINS4 | 4.160 | LOC478498 | 5.115 | FBXL18 | 2.113 |
| TC64242 | 2.353 | TC59609 | 2.205 | TC55499 | 5.514 | TCN1 | 9.329 | NUP107 | 2.520 |
| A_11_P061966 | 5.925 | TC58852 | 2.190 | CO683592 | 2.012 | EXOSC4 | 2.044 | HSPA4L | 3.223 |
| DTL | 5.770 | UCHL1 | 9.267 | ATAD2 | 3.428 | CDC2 | 10.666 | CHEK2 | 2.325 |
| CASC5 | 6.873 | DIAPH3 | 7.278 | TC56683 | 2.071 | TC74646 | 3.487 | A_11_P097186 | 5.233 |
| CCNE1 | 2.780 | A_11_P113751 | 4.867 | ASPM | 4.958 | CO587007 | 3.757 | GADD45GIP1 | 2.239 |
| TC78287 | 2.733 | PSMC3IP | 2.094 | KIF20A | 3.745 | CO598693 | 2.056 | TC69524 | 4.617 |
| MYBL2 | 5.030 | DN386776 | 2.394 | A_11_P068536 | 2.987 | CO671033 | 2.075 | S100A9 | 22.083 |
| TC57951 | 2.603 | BRCA2 | 2.511 | SAG | 7.431 | CCDC99 | 2.444 | DN866325 | 2.216 |
| FANCI | 2.502 | ORC6L | 6.824 | FGF14 | 3.376 | PCNA | 2.756 | TC62757 | 2.803 |
| TC47900 | 2.327 | P2RX3 | 2.240 | SFXN1 | 2.378 | RSPH10B | 2.744 | A_11_P0000032564 | 2.935 |
| TC70337 | 2.959 | ENSCAFT00000006773 | 2.600 | A_11_P0000031120 | 2.496 | PCDHGA3 | 2.737 | S100A9 | 20.853 |
| SPAG5 | 7.265 | KIFC1 | 6.984 | RAD23A | 2.113 | CKAP2L | 8.921 | A_11_P058641 | 2.347 |
| A_11_P0000030038 | 7.205 | MCM3 | 3.152 | CARHSP1 | 3.122 | DSCC1 | 2.028 | LOC491556 | 5.169 |
