# Supplementary material for: Global Gene Expression Analysis of Canine Cutaneous Mast Cell Tumor: Could Molecular Profiling Be Useful for Subtype Classification and Prognostication?
Source: PLoS One. 2014 Apr 18;9(4):e95481. doi: 10.1371/journal.pone.0095481 (PMC3991658; doi:10.1371/journal.pone.0095481)
Supplement: Table S5 — List of selected genes: Ensembl Genome Browser sequence ID, primer sequences, UPL probe and amplicon size. The table defines target and internal control genes selected for qPCR analysis and all the information about qPCR assay design: transcript Ensembl Genome Browser ID, primer sequences, UPL probe and amplicon size. (DOCX) [file pone.0095481.s005.docx]

**Table S5.** List of selected genes: Ensembl Genome Browser sequence ID, primer sequences, UPL probe and amplicon size.

| **Gene** | **Gene description** | **Ensembl Genome Browser sequence ID** | **Primer sequence (5’-3’)** | **UPL probe** | **Amplicon size**  **(bp)** |
| --- | --- | --- | --- | --- | --- |
| CCNB2 | Cyclin B2 | [ENSCAFT00000026290](http://www.ensembl.org/Canis_familiaris/Transcript/Summary?t=ENSCAFT00000026290) | F: CCAGTACAGATGGAAATGTTGG R: AGGTTCTCTTCCTTCATGGAGAT | #81 | 77 |
| CDC20 | Cell division cycle 20 | [ENSCAFT00000008495](http://www.ensembl.org/Canis_familiaris/Transcript/Summary?db=core;g=ENSCAFG00000005259;r=15:16798320-16806713;t=ENSCAFT00000008495) | F: GTGCCGTGGATGCTCAAT R: CCAGAGATGAGCTCCTTGTAGTG | #54 | 70 |
| CDCA8 | Cell division cycle associated 8 | [ENSCAFT00000005257](http://www.ensembl.org/Canis_familiaris/Transcript/Summary?t=ENSCAFT00000005257) | F: GGTTTGACTCAAGGGTCTTCA R: GAGATGTTGTAGATCCGCTCTCT | #3 | 73 |
| CENPP | Centromere protein P | [ENSCAFT00000003607](http://www.ensembl.org/Canis_familiaris/Transcript/Summary?t=ENSCAFT00000003607) | F: TTCCGAAGCTGGATCTTCTC R: AGTCTCCAGGATGTGGTGCT | #1 | 74 |
| FEN1 | Flap structure specific endonuclease 1 | [ENSCAFT00000049322](http://www.ensembl.org/Canis_familiaris/Transcript/Summary?t=ENSCAFT00000049322) | F: AGGAGCAATTTGTAGATCTGTGC R: CCCGAATGCTCTCACAGTAGT | #3 | 60 |
| FOXM1 | Forkhead box M1 | [ENSCAFT00000024793](http://www.ensembl.org/Canis_familiaris/Transcript/Summary?t=ENSCAFT00000024793) | F: CGAGGATCACTTCCCCTATTT R: GAAAGGTTGTGGCGGATG | #11 | 72 |
| GSN | Gelsolin | [ENSCAFT00000005907](http://www.ensembl.org/Canis_familiaris/Transcript/Summary?db=core;g=ENSCAFG00000003663;r=11:74248247-74268129;t=ENSCAFT00000005907) | F: CCTGGGACAGCTTCAACAAC R: CCGCACCACTGGTAGATGT | #61 | 70 |
| KPNA2 | Karyopherin alpha 2 | [ENSCAFT00000018435](http://www.ensembl.org/Canis_familiaris/Transcript/Summary?t=ENSCAFT00000018435) | F: TGTCAAAGGCATAAATAGCAACA R: AGCAGTTTCCTAGCAGCTTGA | #1 | 69 |
| NUF2 | NDC80 kinetochore complex component, homolog (S. cerevisiae) | [ENSCAFT00000021052](http://www.ensembl.org/Canis_familiaris/Transcript/Summary?t=ENSCAFT00000021052) | F: ATGAAAGACACAGTCCAGAAACTT R: GCAAGCAATCAACGGAGTCT | #137 | 89 |
| NUSAP1 | Nucleolar and spindle associated protein 1 | [ENSCAFT00000015151](http://www.ensembl.org/Canis_familiaris/Transcript/Summary?t=ENSCAFT00000015151) | F: GCCTACCTTAAAGACGAAGCAA R: CATCTGCATCTCAGTCTCATCC | #88 | 93 |
| PRC1 | Protein regulator of cytokinesis 1 | [ENSCAFT00000019302](http://www.ensembl.org/Canis_familiaris/Transcript/Summary?t=ENSCAFT00000019302) | F: CAACGAGCCAAGCTTCAAA R: ATGTTTCAATCCGTGCCTTC | #144 | 67 |
| RAD51 | DNA repair protein RAD51 homolog 1 (S. cerevisiae) | [ENSCAFT00000014658](http://www.ensembl.org/Canis_familiaris/Transcript/Summary?t=ENSCAFT00000014658) | F: GGCCATGTACATTGACACTGA R: CACTGCCAGAGAGGCCATA | #102 | 83 |
| UBE2S | Ubiquitin-conjugating enzyme E2S | [ENSCAFT00000045087](http://www.ensembl.org/Canis_familiaris/Transcript/Summary?t=ENSCAFT00000045087) | F: GCATGTCCTGCTGACCATC R: CTCATTGAGGGCCGACTC | #40 | 64 |
| ATP5B | ATP synthase subunit beta, mitochondrial | [ENSCAFT00000000224](http://www.ensembl.org/Canis_familiaris/Transcript/Summary?t=ENSCAFT00000000224) | F: TCTGAAGGAGACCATCAAAGG R: AGAAGGCCTGTTCTGGAAGAT | #120 | 74 |
| TMBIM4 | Transmembrane BAX inhibitor motif containing 4 | [ENSCAFT00000000579](http://www.ensembl.org/Canis_familiaris/Transcript/Summary?t=ENSCAFT00000000579) | F: TCTACAATCTAAGAGAGATTTCAGCAA R: TTCCTGACAAGCACAAAATCC | #15 | 77 |
| HPRT1 | Hypoxanthine-guanine Phosphoribosyl transferase 1 | [ENSCAFT00000045759](http://www.ensembl.org/Canis_familiaris/Transcript/Summary?t=ENSCAFT00000045759) | F: TGCTCGAGATGTGATGAAGG R: TCCCCTGTTGACTGGTCATT | #62 | 192 |
