# Supplementary material for: Global Gene Expression Analysis of Canine Cutaneous Mast Cell Tumor: Could Molecular Profiling Be Useful for Subtype Classification and Prognostication?
Source: PLoS One. 2014 Apr 18;9(4):e95481. doi: 10.1371/journal.pone.0095481 (PMC3991658; doi:10.1371/journal.pone.0095481)
Supplement: Table S6 — qPCR assay parameters: primer concentration, efficiency, error value and dynamic range. The table defines the main features of qPCR assays obtained during their setting up. In particular primer concentration and efficiency, error value and dynamic range are reported. (DOCX) [file pone.0095481.s006.docx]

**Table S6.** qPCR assay parameters: primer concentration, efficiency, error value and dynamic range.

| **Gene** | **Primers concentration** | **Efficiency**  **(%)** | **Error value*** | **Dynamic range (CP)** |
| --- | --- | --- | --- | --- |
| CCNB2 | 600F/600R | 105.0 | 0.004 | 26.79 – 36.94 |
| CDC20 | 600F/600R | 96.0 | 0.028 | 27.19 – 36.33 |
| CDCA8 | 600F/600R | 98.1 | 0.009 | 26.24 – 36.19 |
| CENPP | 600F/600R | 94.9 | 0.012 | 33.53 – 40.00 |
| FEN1 | 300F/300R | 102.9 | 0.006 | 24.77 – 33.77 |
| FOXM1 | 600F/600R | 98.6 | 0.008 | 25.85 – 35.49 |
| GSN | 600F/600R | 96.8 | 0.005 | 21.12 – 32.59 |
| KPNA2 | 300F/300R | 96.8 | 0.004 | 23.62 – 35.27 |
| NUF2 | 600F/600R | 99.6 | 0.010 | 26.57 – 37.74 |
| NUSAP1 | 600F/600R | 100.5 | 0.016 | 24.34 – 35.66 |
| PRC1 | 300F/300R | 96.5 | 0.007 | 24.22 – 35.45 |
| RAD51 | 600F/600R | 95.3 | 0.021 | 26.58 – 35.95 |
| UBE2S | 300F/300R | 98.9 | 0.013 | 24.84 – 34.67 |
| ATP5B | 600F/600R | 104.2 | 0.008 | 20.48 – 31.37 |
| CGI-119 | 600F/300R | 93.0 | 0.011 | 23.77 – 35.23 |
| HPRT1 | 300F/600R | 93.0 | 0.009 | 23.56 – 33.46 |

F: forward primer; R: reverse primer.

*: mean squared error of the single data points fit to the regression line
